# The sex effects on changes in jump performance following an isometric back squat conditioning activity in trained adults

**DOI:** 10.3389/fphys.2023.1156636

**Published:** 2023-04-13

**Authors:** Dawid Koźlenia, Jarosław Domaradzki

**Affiliations:** Unit of Biostructure, Faculty of Physical Education and Sport, Wroclaw University of Health and Sport Sciences, Wroclaw, Poland

**Keywords:** jump height, power, males, females, post-activation performance enhancement, isometry, squat, countermovement jump

## Abstract

There are limited data concerning the disparity between males and females in post-activation performance enhancement (PAPE) based on isometry. Therefore, this study aimed to establish if sex differences exist in the PAPE effect on jump height. The study included 30 males and 15 females aged between 19 and 25, with relative strength in the back squat of at least 110% of body weight and a minimum of 3 years of resistance training experience. A baseline countermovement jump (CMJ) was performed, and the PAPE protocol, which involved three 4-s sets of isometric full-back squats with a 1-min rest interval, was introduced. Five CMJs were performed over the following 9 minutes in 2 minutes rest intervals. Changes (Δ) towards the baseline and each jump height results were calculated and analyzed in the absolute (cm) and relative (%) approach. The repeated measures ANOVA with sex as between-groups effect and time of the changes as within-group effect were conducted. Results showed statistically significant interaction (sex×time) in absolute changes (Δ cm) (F = 2.50, η^2^ = 0.05, *p* = 0.0447), which indicated that the sex effect has changed over time. Post-hoc test showed that during the first 3 minutes, men and women benefited equally, but in the fifth and seventh minutes, the observed changes were greater in men, thus close to significance (*p* = 0.0797, *p* = 0.0786), and in the last minute, the difference was statistically significant (*p* = 0.0309). Also, a statistically significant interaction effect was observed for relative changes (Δ %) (F = 4.22, η^2^ = 0.09, *p* = 0.0027). At the beginning (the first and third minutes), changes in females were greater than in males, but the differences were insignificant. However, after 5 minutes, the decrease in females was observed with statistically significant differences in the last minute compared to males (*p* = 0.0391). Chi-Squared analysis indicated that the time to peak performance was insignificant (*χ* ^*2*^ = 7.45, *p* = 0.1140) in both sexes. The introduced PAPE protocol based on isometry improved jump height in both sexes, with performance enhancement recorded in the third-minute post-activation. However, performance decreased in females over the next 6 minutes, while it was maintained in the male group. Despite the generally positive short-term effects of the protocol on females, the usefulness of the protocol is limited.

## 1 Introduction

Post-activation performance enhancement (PAPE) can be achieved using various methods, though the essential criteria for peak performance and the avoidance of fatigue include proper set intensity and rest intervals. Furthermore, it is important to use a conditioning activity (CA) with a movement pattern related to the target effort, such as a squat for a jump (Seitz and Haff, 2016; Blazevich & Babault, 2019; Boullosa, 2021). Many authors have developed effective conditioning protocols based on dynamic and isometric actions, although the latter is rarely used (Rixon et al., 2007; Guerra et al., 2022; Pereira et al., 2022; Đurović et al., 2022).

Isometry has many benefits associated with less energy expenditure, meaning that fatigue is limited and neural drive is improved, which may enhance performance (Duchateau and Hainaut, 1984; Baudry and Duchateau, 1985; Cady et al., 1989). Indeed, post-activation enhancement protocols based on isometry (have positively impacted performance in several studies (Rixon et al., 2007; Hirayama, 2014; Spieszny et al., 2022), although others have shown them to have no benefits (Harat et al., 2020). In this regard, it is necessary to emphasize that many factors other than the introduced protocol can influence the PAPE effect. Therefore, individualizing the protocol and adjusting for various cofactors is essential (Seitz and Haff, 2016).

It is difficult to establish from the results of PAPE studies which type of muscle contraction is superior, although it is well-known that personal factors such as baseline strength and sports experience can influence PAPE (Seitz and Haff, 2016; Dobbs et al., 2019). Also, unilateral CA may enhance athletic performance (Krzysztofik et al., 2022). However, limited studies directly compare PAPE effects between males and females, and the results are conflicting (Boullosa, 2021). Non-etheless, studies have shown a positive impact of PAPE on males and females in various sports disciplines. A study by Pereira et al. (2022) demonstrated performance improvement among male sprinters after introducing a and Matusiński et al. (2021) showed improvement in sprint performance in females after a PAPE protocol. Meanwhile, PAPE improved jump performance for both sexes involved in team sports (Masel & Maciejczyk, 2022; Villalon-Gasch et al., 2022). Despite these studies adopting various protocols, their observations demonstrate that achieving a positive effect on performance in both sexes is possible. Considering the previously mentioned observations, there is a lack of consensus if using the same PAPE protocol could provide similar results in both sexes. Previously Rixon et al. (2007) revealed improved power output and jump height after an isometric CA, with better improvement seen in males and similarly observed Arabatzi et al. (2014) with no significant changes in women. However, research by Tsolakis et al. (2011) showed a decrease in power parameters in males after isometry CA with no changes in women. Based on these studies, it is difficult to conclude if there is a need to tailor PAPE protocols concerning sex. Moreover, most previous studies compared direct jump height results, not adjusted to baseline differences in jump height regarding sex. Therefore, there is a need to analyze the PAPE effect between sexes as a change (Δ) in performed measures to establish real differences in the effects of introduced CA.

There are limited data evaluating sex effects on PAPE outcomes (Boullosa, 2021). As such, questions over whether using the same protocol in males and females leads to similar results or if the protocol needs to be adjusted for sex remain unanswered, especially regarding using isometry activity. Therefore, this study aimed to establish if sex differences exist in the PAPE effect based on isometric effort. This was achieved by posing three specific questions: (1) does a PAPE protocol based on isometry lead to changes in jump height in males and females?; and (2) how do these results differ regarding sex?; (3) additionally, when does the peak performance improvement occur? The results will provide more insight into the PAPE phenomenon and establish if CA protocols based on isometry should be adjusted for sex.

## 2 Material and methods

### 2.1 Study design

The experiment consisted of two meetings. At the first meeting, the participants provided information about their health status, training experience, and musculoskeletal injury history. Body morphology measurements were also recorded, participants’ full-back squat one-repetition maximum (1-RM) was established, and they were familiarized with the testing methods. Participants performed a standard warm-up at the second meeting, and a countermovement jump (CMJ) baseline jump height was recorded. The PAPE protocol was introduced, and five CMJ measurements were taken after 1, 3, 5, 7, and 9 min.

### 2.2 Participants

This study was approved by the Ethics Committee of the Wroclaw University of Sport Health Sciences (16/2018) and was conducted according to the guidelines of the Declaration of Helsinki. Participants were informed about the study procedure and possible risks of the experiment. All of them were volunteers and provided written informed consent agreeing to the conditions of the study. They were allowed to exit the study at any moment.

Ninety-three subjects were identified as potential study participants. The inclusion criteria were age 19–25 years old, no musculoskeletal injury 8 weeks before the study, no other medical contradictions, experience in strength training of at least 3 years, at least 1 year in continuous resistance training, the ability to perform a back squat with 110% of body weight, and no use of any doping substances such as anabolic-androgenic steroids. Forty-five participants qualified for the main stage of the experiment, including 30 males and 15 females. Participants were asked to avoid intense physical effort for 48 h before measurements and maintain normal eating, drinking, and sleeping habits. However, ergogenic compounds such as caffeine were not to be consumed in the 24 h before the measurements. A detailed description of the participants is presented in the results section.

### 2.3 Body morphology

Body height was measured with a Swiss anthropometer (GPM Anthropological Instruments, DKSH Ltd., Zürich, Switzerland), and body mass was assessed with an InBody230 device (InBody Co., Ltd., Cerritos, CA, United States), which has been confirmed to be highly reliable (McLester et al., 2020). Based on the obtained data, Body Mass Index (BMI) was calculated using the following formula:

$$BMI=body\;mass\left[kg\right]/body\;height\left[m^{2}\right]$$

### 2.4 Full-back squat one maximum repetition testing

Using the Vitruve velocity-based training (VBT) device (Vitruve, SPEED4LIFTS S.L., Madrid, Spain), the 1-RM for the full-back squat was established based on the association between load and velocity (Jidovtseff et al., 2011; Signore, 2021), ensuring an exact and safe method. The device’s reliability was previously confirmed (Martínez-Cava et al., 2020). Participants performed a standard warm-up consisting of a 5-min run at 6 km/h, dynamic joint mobilization, and two rounds of ten repetitions of body squats, lunges, and hip thrusts with a 60-s break between each round. After the general warm-up, a specific routine was introduced. Firstly, the participants performed a maximal deep-back squat with an empty bar to establish the maximal individual depth of the back squat while maintaining proper technique and a safe body position. The same maximum back squat depth was used in every repetition of the 1-RM protocol and during the CA and was controlled by parallels set corresponding to height. The investigator was always present to ensure repeatability was maintained through the proper performance of every set.

The warm-up set started with 12–15 repetitions with an empty bar, then 10 to 12 repetitions at 50%-RM (mean velocity of 1.0–1.2 m/s) after two to 3 minutes. Subsequently, participants performed two to three sets of five repetitions at 60–80%-RM (mean velocity of 0.5–0.75 m/s). After achieving the demand velocity, a progressive incremental test was performed. An initial load of 80%-RM was used for three repetitions, with 5%–10% load increases. When the mean velocity decreased below 0.5 m/s during the set, the next set was performed for two repetitions, and one repetition was performed when it fell below 0.25 m/s. The test was performed for a maximum of five sets or until failure occurred, with three to 5-min breaks allowed between sets.

### 2.5 Countermovement jump

CMJ height was measured using the OptoJump device (Microgate, Bolzano, Italy) and was calculated at a frequency of 1,000 Hz using the following equation:

$$Jump\;height=9.81\times \left[flight\;time\right]2/8$$

The validity and reliability of the OptoJump device were previously confirmed (Słomka et al., 2017; Rago et al., 2018). The CMJ was performed without arm swings, with no depth restrictions. Six measurements were taken for analysis, including baseline (before the CA) and 1, 3, 5, 7, and 9 min after the CA. Based on the previous studies, peak performance should be achieved in the relevant time (Guo et al., 2023; Bogdanis et al., 2014; Wilson et al., 2013; Dobbs et al., 2019).

### 2.6 Conditioning activity

**FIGURE 1:**
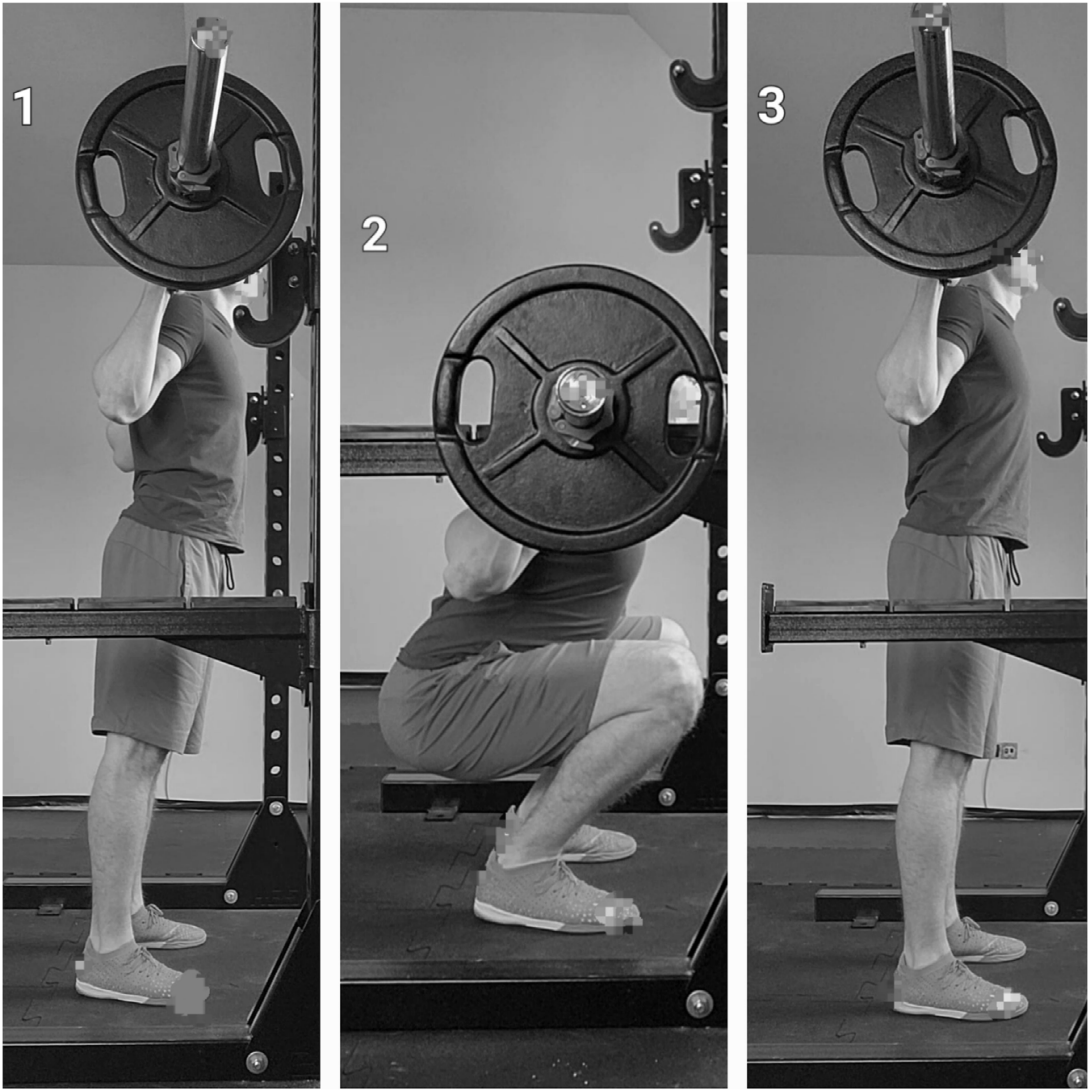
Conditioning activity. 1—starting position; 2—isometry hold; 3—final position.

The participants performed a standard warm-up, which included 5 minutes of running on a treadmill at 6 km/h. They then performed dynamic joint mobilization and two rounds of 10 repetitions of body squats, lunges, and hip thrusts, with a 60-s break between rounds. Participants then completed three CMJs, with a 30-s rest between trials and the warm-up. After a 3-min rest, the baseline CMJ was recorded. Participants then performed an activation protocol based on isometric effort, including three sets with 60-s breaks between them of full-back squats at 70%-RM. Individuals achieved the deepest position (established during the familiarization session) by maintaining a proper technique bends his knees and lowershis torso until when the upper surface of the thighs at the hip joint is lower than the tops of the knees and stopping the movement for 4 seconds (number 2 on the graph). See Figure 1. The spotters were always present. CMJ measurements were recorded following these activities.

### 2.7 Statistics

Power analysis was conducted to determine the required sample size using GPower version 3.1.9.6 software (Heinrich Heine University, Düsseldorf, Germany). Assuming 95% power and a minimum effect size (ES) of 0.25 at *α* = 0.05, the minimal sample size required to detect changes in the measured parameters was 32 subjects.

Statistica 13.0 software (StatSoft Poland, Cracow, Poland) was used for the analysis, and results are expressed as mean ± standard deviation (SD) and 95% confidence intervals. The CMJ measurements were validated by assessing test-retest reliability using the intraclass correlation coefficient (ICC 2.1) criteria set by Koo & Li (2016). Data distribution was examined for normality using the Shapiro-Wilk, and the Levene test was performed to evaluate variance homogeneity between groups. The sphericity assumption for the repeated measures was examined with Mauchly’s test of sphericity. Then, repeated measures ANOVA with sex as a between-group effect and time (in minutes of the CMJs performing) as a within-group effect was performed to assess the changes (Δ) in jump height results after PAPE protocol. The analyzed data was an absolute change (Δ cm) and relative change (Δ %) calculated based on the differences between the baseline and results from the following minutes of measurements. Then Duncan’s *post hoc* test was performed to investigate detailed differences between measures. A Chi-Squared (*χ* ^*2*^) statistic was calculated to assess if the frequency of time the peak occurred after conditioning activity (best jump height) was significant. The significance level was set at *p* < 0.05.

## 3 Results

The reliability of the method used to measure CMJ height was assessed individually for each sex. Males had an ICC 2.1 = 0.90, whereas females had an ICC 2.1 = 0.92, indicating that the results obtained for the CMJ measurements are credible. Detailed characteristics of the study participants are presented in Table 1.

**TABLE 1:** Participants characteristics.

Variable	Men	Women
	Mean ± SD (CI95%)	Mean ± SD (CI95%)
Age [years]	21.2 ± 1.7 (20.6–21.9)	21.2 ± 2 (20.1–22.3)
Body height [m]	1.8 ± 0.1 (1.8–1.8)	1.7 ± 0.1 (1.7–1.7)
Body weight [kg]	79.5 ± 8.8 (76.2–82.8)	60.5 ± 7.1 (56.6–64.4)
BMI [kg/m2]	24.4 ± 2.1 (23.7–25.2)	20.8 ± 1.8 (19.9–21.8)
Gym experience [years]	4.3 ± 1.9 (3.6–5)	4.6 ± 1.7 (3.7–5.5)
Relative strength [bw%]	1.5 ± 0.3 (1.4–1.6)	1.2 ± 0.1 (1.1–1.3)
CMJ baseline [cm]	36.9 ± 6 (34.6–39.1)	23.7 ± 4.2 (21.4–26.1)
Δ cm Baseline -1′	0.7 ± 2.3 (−0.2–1.5)	0.5 ± 1.7 (−0.4–1.5)
Δ cm Baseline-3′	1.6 ± 2.3 (0.8–2.5)	1.4 ± 1.9 (0.3–2.5)
Δ cm Baseline-5′	1.6 ± 2.3 (0.8–2.5)	0.4 ± 1.7 (−0.6–1.3)
Δ cm Baseline-7′	1.7 ± 2 (0.9–2.4)	0.3 ± 2 (−0.8–1.4)
Δ cm Baseline-9′	1.3 ± 2 (0.6–2.1)	−0.3 ± 1.9 (−1.3–0.8)
Δ% in 1′	1.7 ± 6.4 (−0.7 - 4.1)	3.1 ± 8.7 (−1.7–7.9)
Δ% in 3′	4.5 ± 6.4 (2.1–6.8)	6.5 ± 9.7 (1.1–11.8)
Δ% in 5′	4.3 ± 6.2 (2–6.6)	2.2 ± 8.7 (−2.6–7)
Δ% in 7′	4.5 ± 5.8 (2.3–6.7)	1.4 ± 9.1 (−3.7–6.4)
Δ% in 9′	3.6 ± 5.3 (1.6–5.6)	−1 ± 8.9 (-6–3.9)

Table 1 presents the descriptive characteristics of the study sample, as well as baseline CMJ height and changes Δ in absolute (cm) and relative approach (%).

The results of the repeated measures ANOVA (with sex, time, and interaction term effects) for absolute changes (Δcm) are presented in Table 2. The analysis revealed a lack of statistically significant effect of sex; thus, it was close (F = 3.06, η^2^ = 0.06, *p* = 0.0876), whereas the main effect of time for absolute changes (Δcm) was statistically significant (F = 3.26, η^2^ = 0.07, *p* = 0.0132), as well the interaction term between both factors was found as statistically significant (F = 2.50, η^2^ = 0.05, *p* = 0.0447). It suggested that the level of the changes in males and females depended on time.

**TABLE 2:** Main effects and interaction effects of sex and time changes in absolute approach (Δ cm). Two-way repeated measures ANOVA.

Effect	F	p	η^2^
Sex	3.06	0.0876	0.07
Time	3.26	0.0132	0.07
Sex×Time	2.50	0.0447	0.05

**FIGURE 2:**
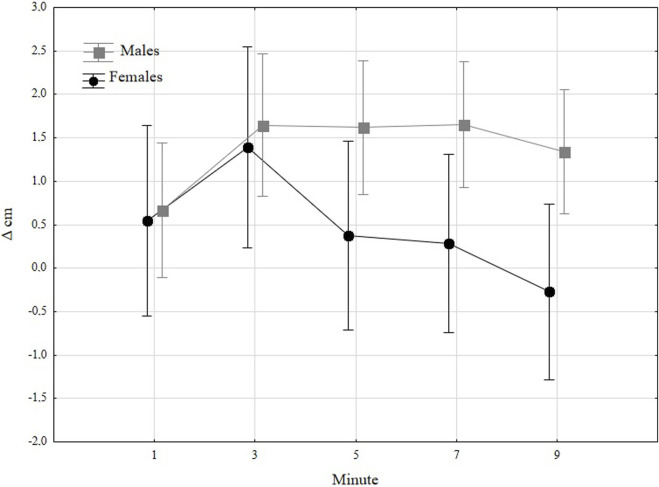
Absolute changes (Δ cm) towards baseline in jump height in males and females.

Figure 2 presents absolute changes (Δ cm) in jump height over time regarding sex. General trends showed improvement among males after 3 minutes which lasted in the following two measurements, with a decrease in the last minute. In women, the peak performance was observed after 3 minutes and then decreased in the following measures. The *post hoc* analysis revealed that changes observed in the third, fifth, and seventh minutes were significantly higher than in the first minute (*p* = 0.0436, *p* = 0.0438, *p* = 0.0454, respectively). In women, the statistically significant improvement was observed in the third minute and was higher than the results observed after five (*p* = 0.0339), seven (*p* = 0.0220), and 9 minutes (*p* = 0.0004). When analyzing between-sex differences, it was visible that after 3 minutes, men and women benefited equally, but after 5 minutes, the differences became clearer, thus in the fifth and seventh minutes, the differences were close to significance (*p* = 0.0797, *p* = 0.0786), and in the last minute the difference was statistically significant (*p* = 0.0309). Table 3.

**TABLE 3:** Main effects and interaction effects of sex and time changes in relative approach (Δ %). Two-way repeated measures ANOVA.

Effect	F	p	η^2^
Sex	0.47	0.4968	0.01
Time	4.76	0.0011	0.10
Sex × Time	4.22	0.0027	0.09

Then, the relative changes in jump height were considered (Δ%). The ANOVA revealed a lack of statistically significant effect of sex (F = 0.47, η^2^ = 0.01, *p* = 0.4968). In contrast, the main effect of time was statistically significant (F = 4.76, η^2^ = 0.10, *p* = 0.0011), as well the interaction term was found as statistically significant (F = 4.22, η^2^ = 0.09, *p* = 0.0027).

**FIGURE 3:**
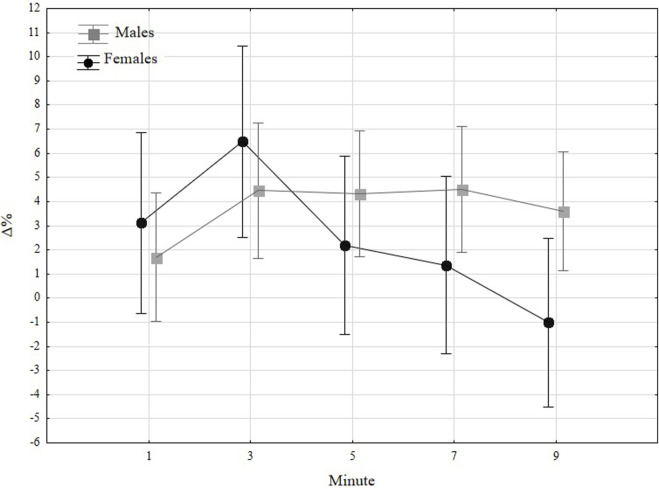
Relative changes (Δ%) towards baseline in jump height in males and females.

Figure 3 states a graphical presentation of relative changes (Δ %) in jump height over time regarding sex. In both sexes, in the beginning, the improvement was greater in women, but after 5 minutes, the decrease in females was observed, whereas, in men, only a slight decline was noticed after 9 minutes. The detailed *post hoc* analysis did not show any significant changes in males; thus, when compared, the change from the first minute to the third minute (*p* = 0.0865), fifth (*p* = 0.0964), and seventh (*p* = 0.0855) minute were close to significant. In women, more changes were significant. The measure from the third minute was higher in the first minute (*p* = 0.0335), fifth (*p* = 0.0062), seventh (*p* = 0.0010), and ninth (*p* < 0.0001). Moreover, the decrease in the last minute was significantly lower than the results observed in the first (0.0070) and fifth minute (0.0357). When considering the between-sex differences, the changes observed in the last minute of measurements were statistically significantly higher in males (*p* = 0.0391).

In the last step, a *χ* ^*2*^ analysis was performed to establish if there was any association between peak performance and time post-activation for either sex. Over half of the females achieved their best results in the first and third minutes (four and five participants, respectively). The peak also occurred in the third minute for males (seven participants) and in the seventh minute (eight participants), indicating that the positive effects occurred faster among females. However, the *χ* ^*2*^ test results were statistically insignificant (*χ* ^*2*^ = 7.45, *p* = 0.1140).

## 4 Discussion

This study aimed to investigate sex differences in post-activation performance enhancement responses by assessing the impact of an isometric CA protocol on jump height. In the initial post-activation period, the jump height increased in both sexes and peaked after 3 minutes. However, analysis of jump height changes in the following minutes revealed different patterns in males and females. The performance enhancement was still seen in males after 9 minutes, whereas females peaked early, and a significant decrease to below baseline levels occurred in jump height at the ninth minute. The obtained results will provide more insight into the PAPE phenomenon and help determine if the same PPE protocols should be adjusted for sex.

PAPE could have significance in sports competitions and training (Boullosa, 2021; Villalon-Gasch et al., 2022; Đurović et al., 2022), although there is still much to discover. Many factors can influence the PAPE effect, various protocols can be utilized (Seitz and Haff, 2016; Dobbs et al., 2019), and greater difficulty can be added to increase variability and individualize the PAPE response (Till & Cooke, 2009). One of the most crucial factors for effective PAPE is appropriate intensity. Indeed, an effort of approximately 80%-RM is submaximal (Seitz and Haff, 2016; Dobbs et al., 2019), though positive effects are observed with higher and lower loads (Gołaś et al., 2017; Vargas-Molina et al., 2021) or by using voluntary contractions (Kalinowski et al., 2022; Spieszny et al., 2022). In this regard, the intensity of the CA and rest intervals are also crucial (Dobbs et al., 2019). In our study, peak performance occurred in the third minute for both sexes and persisted over the following 6 minutes in males. It is in line with the study by Guo et al. (2023), which demonstrates that peak performance occurred 3 minutes after introduced PAPE protocol; Bogdanis et al. (2014) investigated the various durations of rest interval (2–21 min) and, similarly to us, found that after the PAPE protocol based on isometry, individuals peaked after 3 minutes. The previously performed meta-analysis by Wilson et al. (2013) also indicated that the best results are observed after 3 min of rest. Still, some individuals could need more time, and it needs to prolongate the rest interval to the next 6 minutes. Lastly, Dobbs et al. (2019) also demonstrate the range of 3–7 min of rest as optimal for peak performance. However, the variability in observations was high when considering individual peak performance. Indeed, despite the mean scores generally indicating when the peak occurred, the results of PAPE are strongly individual regardless of protocol type (Gouvêa et al., 2013; Evetovich et al., 2015).

This study did not show a statistically significant sex effect when assessing the time taken to reach peak performance. However, females generally achieved their best jump height after 3 minutes, whereas males achieved their best results later. These results highlight the importance of individual responses in PAPE (Wilson et al., 2013).

French et al. (2003) showed positive effects of three to 5 seconds of isometric contractions on dynamic efforts. In the current study, a protocol of three 4-s sets of isometric deep squats also provided positive results in jump height. Vargas-Molina et al. (2021) also observed jump improvement after isometric squats, though they used a slightly higher external load. In addition, Spieszny et al. (2022) employed an isometric squat with an immovable bar to improve jump height and demonstrated that volitional isometric contraction could increase jump height. Tillin and Bishop (2009) postulated that isometric effort might engage more high-threshold motor units and is less fatigue-resistant, indicating that caution is needed when isometry is used. The optimal duration of effort seems to be between three and 5 seconds (French et al., 2003).

The similarity in movement patterns between the CA and targeted effort is an important factor to consider. As such, many studies use quarter-squats or half-squats before jump testing (Dobbs et al., 2016), which seems to be effective when dynamic contraction is demanded (Loturco et al., 2022). However, Esformes and Bampouras (2013) indicated that a full range of motion leads to increased motor unit activation. In this regard, adjusting the depth of the squat according to individual ability may prove beneficial and was employed in the current study. Indeed, using a half-squat or quarter-squat may not be natural for the investigated individuals and may influence the PAPE effects.

The main aim of this study was to analyze sex differences in outcomes following a PAPE based on isometry protocol. The absolute and relative improvements were comparable in both sexes when considering the best post-activation results. However, a more detailed analysis indicated a much shorter effect in females, which suggests that a sex-specific approach is required. Many studies have shown positive PAPE effects in males and females, meaning that the PAPE phenomenon occurs in both sexes (Matusiński et al., 2021; Masel & Maciejczyk, 2022; Pereira et al., 2022; Villalon-Gasch et al., 2022). Non-etheless, few studies have directly considered sex differences in PAPE. Rixon et al. (2007) observed that a PAPE protocol based on isometry evoked better jump enhancement in males than in females, which aligns with our observations. In contrast, Tsolakis et al. (2011) did not find any positive effects after isometric activity in elite fencers and instead showed a deterioration in power output in male athletes. However, the results of Arabatzi et al. (2014) are in agreement with our observations and indicate that males generally benefit more from PAPE protocols. A possible explanation may be that fatigue occurs with a high load, meaning that caution should be taken when PAPE protocols are utilized in females. In this regard, there are biological differences between males and females in body tissue composition and muscle architecture, which leads to differences in motor ability (Miller et al., 1993; Bartolomei et al., 2021). Despite using similar loads and having similar training experiences, males are stronger than females (Bishop et al., 1987). Indeed, even using the individualized relative load in females resulted in an external load that was too high to maintain jump enhancement during testing. The load used led to a brief improvement in jump performance, though it was followed by a decrease in performance. Such a response negates the usefulness of the proposed CA procedure in a real-world setting. Therefore, there is a need to individualize external loads and assess if lighter loads would be more suitable for females.

The study was limited by the unequal number of participants in the groups. Non-etheless, this is one of only a few studies considering sex differences in PAPE based on isometry and provides a more comprehensive picture of the phenomenon.

## 5 Conclusion

The isometric squat-based PAPE protocol could improve jump height in both sexes. The peak performance occurred in females and males in the third-minute post-activation. However, the effect persisted for a shorter period in females as performance fell below baseline levels at the ninth minute, but the improvement was still present in males. Therefore, there is a need to optimize the effort intensity to reduce fatigue in females. There were also differences in the time to achieve peak performance between the sexes. Females peaked after the first 3 minutes post-activation, while the effect appeared later in males. This indicates the need for a shorter break interval after the CA for females and a longer interval for males. There is also a need to individualize the PAPE protocol irrespective of sex, as group variability was observed. Despite the generally positive short-term effects of the PAPE protocol on females, its usefulness in sports competitions is limited. Therefore, there is a need to establish an optimal CA for females that considers the intensity of the external load and the rest interval. This should limit fatigue and enhance the activation effect over a more extended period.

## 6 Practical application

When an improvement in jump height is required, a PAPE protocol based on three 4-s sets of isometric deep squats with 60-s rest intervals can be introduced for males. Generally, peak performance occurred in the third minute and persisted for the next 6 minutes. The data also indicated the possibility of establishing optimal CA protocols individually. Peak performance also appeared in the third minute among females, but the short-term nature of the improvement means that the proposed protocol is of limited use in this group. In particular, caution is advised due to the decrease in jump performance in the extended post-activation period.

## Data Availability

The raw data supporting the conclusions of this article will be made available by the authors, without undue reservation.
